# Advancements in Nanotechnology‐Based PEDOT and Its Composites for Wearable Thermoelectric Applications

**DOI:** 10.1002/smsc.202400149

**Published:** 2024-08-07

**Authors:** Yuran Wang, Wei Dai, Tian Wu, Hongyan Qi, Junhui Tao, Chuanhui Wang, Jie Li, Xiuying Cao, Liangpeng Liu, Liuyi Fang, Chun Wang, Nengyuan Gong, Yuxuan Liu, Xinqi Chen, Wan Jiang, Xiaolin Wang

**Affiliations:** ^1^ School of Physics and Mechanical & Electrical Engineering Hubei Engineering Technology Research Center of Environmental Purification Materials Hubei University of Education Wuhan 430205 China; ^2^ State Key Laboratory for Modification of Chemical Fibers and Polymer Materials Donghua University Shanghai 201620 China; ^3^ Institute for Superconducting & Electronic Materials (ISEM) University of Wollongong Innovation Campus North Wollongong NSW 2500 Australia

**Keywords:** nanotechnology, poly(3,4‐ethylenedioxythiophene), thermoelectric material, thermoelectric properties, wearable technology

## Abstract

Thermoelectric materials’ unique merits attract considerable attention. Among those merits, the straight transformation between heat and electricity makes this material potential. The energy of the human body is released in the form of heat, which can be transformed into effective electricity by wearable thermoelectric materials. The nanotechnology‐based materials improve thermoelectric properties and heat absorption abilities for nanostructures will help maintain good electrical conductivity and reduce thermal conductivity. Poly(3,4‐ethylenedioxythiophene) (PEDOT) is extensively investigated for its high conductivity, flexibility, good transparency, and so on. This article reviews its mechanism and describes the preparation techniques and thermoelectric properties of nanotechnology‐based PEDOT, inorganic semiconductor composite, and low‐dimensional metal composite thermoelectric materials. The recent research progress on PEDOT‐based thermoelectric materials, the application of wearable low‐dimensional PEDOT‐based thermoelectric materials, and methods to improve the thermoelectric performance of PEDOT‐based composite materials, device design, and commercialization are specifically discussed.

## Graphical abstract

This review discusses the thermoelectric properties and preparation techniques of low‐dimensional PEDOT composites, including carbon nanocomposites, inorganic semiconductor composites, and metal composites. It also highlights the application potential of PEDOT in flexible, wearable thermoelectric devices, and discusses methods to enhance their performance, with a focus on device design and commercialization.

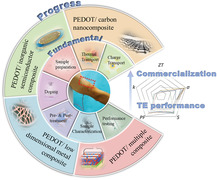

## 1 Introduction

Thermoelectric materials enable the mutual conversion between thermal energy and electrical energy reality and achieve the reuse of waste heat.

Most of the human body's energy is released in the form of heat with an average temperature difference of 5 to 30 Celsius in the environment.^[^ ^1^ ^]^ The wearable thermoelectric materials can convert this low‐grade energy emitted by the human body into useful electrical energy,^[^ ^2^ ^]^ which has the potential to provide adequate energy for some low‐power wireless sensor nodes, while also possessing characteristics such as non‐pollution, portability, and stability, making scientists increasingly attracted.^[^ ^3^ ^]^

These days, researches on flexible wearable thermoelectric generators mainly focus on three types: bulk‐type thermoelectric materials,^[^ ^4^ ^]^ film‐type thermoelectric materials,^[^ ^4^ ^]^ and textile‐type thermoelectric materials.^[^ ^5^ ^]^ The output power of bulk thermoelectric generators is generally tens of micro‐watts per square centimetre and possesses good electrical and thermoelectric properties. The concerning researches focus on improving the output performance and flexibility of such bulk‐type thermoelectric devices.^[^ ^5^ ^]^ Film‐type thermoelectric generators have an output power between nano‐watts and micro‐watts per square centimetre and are able to be classified as horizontal or vertical depending on their structure.^[^ ^4^ ^]^ Common horizontal devices include series, stacked and rolled‐up, and folded types, which usually generate a higher output voltage, while vertical devices have a higher temperature figure of merit due to the increased number of thermoelectric legs per unit area, resulting in a higher power density.^[^ ^6^ ^]^ Textile‐type thermoelectric generators have a low output power but exhibit excellent stretchability, bending, and in‐plane shear performance, making them suitable for 3D deformation and better suited for collecting heat on the curved surface of the skin.^[^ ^7^ ^]^

In recent decades, numerous studies have been carried out to understand the properties of bulk‐type and film‐type thermoelectric materials, while the improvement of a thermal electric figure of merit (*ZT*) has made rapid progress, with many bulk‐type thermoelectric materials’ maximum *ZT* values exceeding 2.^[^ ^8^ ^]^ Low‐dimensional thermoelectric materials exhibit superior thermoelectric properties compared to bulk materials due to enhanced density of states near the Fermi level from quantum confinement, which boosts thermopower, and effective scattering of phonons by dense interfaces, reducing lattice thermal conductivity.^[^ ^9^ ^]^ However, there is still a significant gap between the thermoelectric figure of merit of these materials fabricated into low‐dimensional nanostructures, such as 2D nanofilms and 1D nanowires, in which thermoelectric properties can be significantly improved.

Poly(3,4‐ethylenedioxythiophene) (PEDOT) is a conductive polymer with wide applications in electronics, flexibility, energy, and sensor fields. Its advantages include high conductivity, flexibility, good transparency, strong corrosion resistance, and ease of processing.^[^ ^10^ ^]^ In textile‐type thermoelectric materials, PEDOT can be printed or coated onto the surface of fibres from conductive films, thereby transforming textiles into devices with thermoelectric effects. PEDOT also has controllable synthesis, and its molecular structure and physical and chemical properties can be adjusted through chemical synthesis methods to further expand its application range. Inorganic thermoelectric materials have excellent thermoelectric properties, but their poor processability and flexibility limit their development in the field of flexible materials. PEDOT can effectively improve its thermoelectric properties by composite with appropriate inorganic materials. For example, PEDOT can be prepared in conjunction with other functional materials such as graphene and nanometals to enhance its thermoelectric performance.^[^ ^11^ ^]^ Therefore, PEDOT has a promising application prospect in textile‐based thermoelectric materials and can be used in self‐luminous textiles, smart temperature‐controlled clothing, and other fields.

This article will elucidate the mechanism of PEDOT‐based thermoelectric materials and describe the preparation techniques and thermoelectric properties of low‐dimensional PEDOT/carbon nanocomposite, inorganic semiconductor composite, and low‐dimensional metal composite thermoelectric materials. It will also review recent research progress on PEDOT‐based thermoelectric materials both domestically and internationally, expound on the application of wearable low‐dimensional PEDOT‐based thermoelectric materials, and finally summarize methods to improve the thermoelectric performance of PEDOT‐based composite materials, device design, and commercialization.

## 2 Fundamentals of Conducting PEDOT

### 2.1 Charge Transport

Thermoelectric generators can generate electricity by harnessing the temperature difference Δ*T* through the Seebeck effect,^[^ ^12^ ^]^ making charge transport a crucial component of the thermoelectric conversion process.^[^ ^13^ ^]^ PEDOT‐based thermoelectric materials have a different charge transport mechanism from conventional inorganic thermoelectric materials. Functioning as a conducting polymer, its π‐conjugated structure plays a critical role in charge transport. The weak intermolecular interactions and thermal stimuli contribute to the dynamic disorder of organic molecules, subsequently influencing the mobility of charge carriers.^[^ ^14^ ^]^ Its band structure is similar to conventional inorganic thermoelectric materials, where the charge transport is determined by conduction/valence bands and band‐gap value. Hence, rational doping can effectively alter the band structure and improve the charge transport of conducting polymers in turn.^[^ ^14^ ^]^

A couple of models have been introduced to investigate the complicated charge transport behaviours of PEDOT‐based thermoelectric materials:

#### 2.1.1 Marcus Model

Also known as a bimolecular reaction. The basic reaction can be described as Equation (1).^[^ ^15^ ^]^

(1) $$D^{-}+A\to D+A^{-}$$

**Figure 1:**
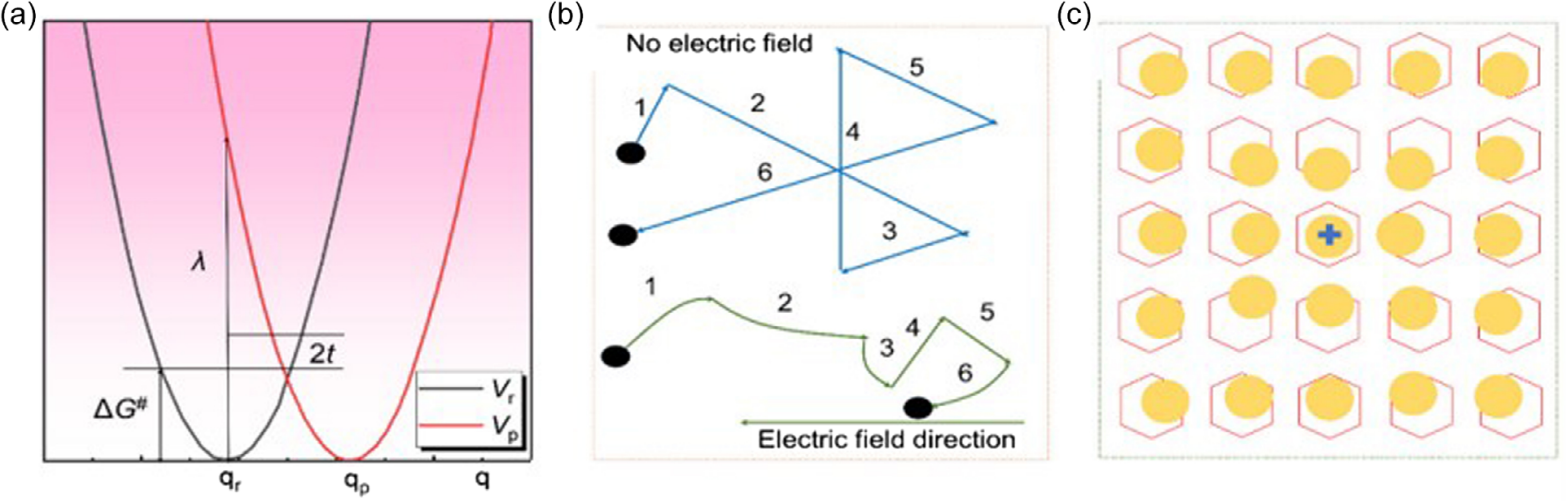
a) Potential energy diagram of charge transfer reaction in conducting polymers. Reproduced with permission.^[^ ^77^ ^]^ Copyright 2007, CRC Press. b) Schematic diagram of the electron transport path in conducting polymers. Reproduced with permission.^[^ ^78^ ^]^ Copyright 2006, Oxford University Press. c) Schematic diagram of the formation of polarons in conducting polymers under the influence of positive charges. Reproduced with permission.^[^ ^77^ ^]^ Copyright 2007, CRC Press.

**Figure** **1**a describes the energy change based on the reaction by the Marcus model, where Δ*G* ^*#*^ is the energy barrier and related to the charge transfer integral (*t*). In this model, Equation (2) and (3) are satisfied.

(2) $$\Delta G^{\#}={\left(\lambda -2t\right)}^{2}{\left(4\lambda \right)}^{-1}=\lambda /4-t\;(t<\lambda )$$

(3) λ=f(qR−qP)22

where *f* is the oscillation intensity, and *q*_*R*_ and *q*_*P*_ are coordinate reactants and products, respectively. Marcus’ theory can be linked to charge transfer speed and carrier mobility *μ*;^[^ ^16^ ^]^

#### 2.1.2 Band‐Like Model

Carriers move freely in the energy gap. In the absence of an external electric field, carriers exhibit solely free thermal motion. However, the introduction of an electric field impels the carriers to migrate in response to the field's influence. Figure 1b shows the carrier transport path with or without an electric field. *σ* and *μ* will decrease if the temperature increase. Band‐like model is suitable for conducting polymers with high intrinsic *σ* and materials with low doping concentration.^[^ ^17^ ^]^

#### 2.1.3 Hopping Model

Forming highly regular crystalline structures in conducting polymers presents a significant challenge, resulting in localized charge movement within amorphous organic matrices through a hopping mechanism. In solid materials, the localized charge becomes “self‐trapped,” leading to noticeable displacements of adjacent atoms. This charge localization gives rise to the formation of polarons accompanied by the induced polarization cloud in the surrounding environment. In conducting polymers, the polarization effect primarily stems from the polarization of the neighbouring π electron cloud, as visually demonstrated in Figure 1c.^[^ ^16^ ^]^

The movement of overloaded carriers driven by the temperature field gives rise to an entropy change known as *S*. This illustrates the core mechanism underlying the thermoelectric conversion, which is influenced by *σ* and *S*. The thermoelectric conversion mechanism reveals that *σ* and *S* have a mutually restrictive relationship, both being directly affected by the carrier concentration (*n*), with np for materials dominated by hole carriers and ne for materials dominated by electron carriers. Interestingly, *S* decreases as n increases, while *σ* exhibits the opposite trend. Furthermore, the electronic thermal conductivity (*κe*) is closely related to n. In the case of conducting polymers, the peaks of these three parameters generally occur within the range of n between 1019 and 1021 cm^−3^. However, the challenge lies in explaining the charge transport mechanism of conducting polymers, as *S* and *σ* are influenced by distinct transport mechanisms, despite researchers’ efforts to develop various transport models. To date, the developed models include: 1) The nearest‐neighbor model is employed to describe the movement of electronic charges from Site A to Site B, while simultaneously allowing other charges to occupy Site A. This model is commonly utilized to explain carrier transport behaviours in heavily doped conducting polymers, especially under thermal excitation. However, it suffers from a notable drawback of low accuracy and limitations in considering site distances;^[^ ^18^ ^]^ 2) The variable‐range hopping model is employed to explain the presence of localized states within a wide energy band, taking into account the energy‐dependent term and determining conductivity (*σ*). This model is particularly suitable for situations involving conductive polymer doping.^[^ ^18^ ^]^ 3) The mobility edge model is suitable for describing charges excited into delocalized states.^[^ ^19^ ^]^ Compared with the hopping model, the mobility edge model matches the conductive polymers with higher *σ*.^[^ ^20^ ^]^ 4) The transport edge model is suitable for some advanced conductive polymers with a higher *σ* similar to metals, which are more able to follow the *S* ∝ *σ* ^1/4^ empirical power law.^[^ ^20^ ^]^ 5) The heterogeneous media model is an appropriate framework for characterizing the disordered regions of conductive polymers with low conductivity (*σ*). However, it is important to note that conductive polymers exhibit a combination of both disorder and crystallinity. They consist of small crystalline domains ranging from 10 to 50 nm, which are interconnected through disordered regions and several extended polymer chains. Therefore, this model is most effective when describing conductive polymers that have sequential sections with both high and low conductivity.^[^ ^21^ ^]^

### 2.2 Thermal Transport

Common organic materials include poly(3,4‐ethylenedioxythiophene): poly(styrene sulfonate) (PEDOT:PSS), poly(3‐butylthiophene) (P3BT), polyaniline (PANI), polyacetylene (PA), polypyrrole (PPy), and polythiophene (PTs), which possess comprehensive characteristics such as low thermal conductivity, lightweight, ease of processing, mechanical flexibility, and environmental friendliness. PEDOT:PSS is essentially a blend of PEDOT and poly(styrene sulfonate) (PSS). PSS is a polymer with negative charges commonly mixed with PEDOT to enhance its dispersibility, stability, and conductivity. Due to its high electrical conductivity, good thermal stability, and low thermal conductivity, PEDOT:PSS is an ideal material for flexible optoelectronic devices. PEDOT:PSS exhibits good stability as an aqueous dispersion and can form stable and continuous thin films on substrates.

The thermal conductivity behavior of PEDOT‐based materials demonstrates intriguing characteristics regarding temperature dependency. While the interchain thermal conductivity remains relatively stable across different temperatures due to the interface‐like heat transfer between PEDOT chains and dopants, the along‐chain and cross‐plane virtual thermal conductivities exhibit a noticeable decrease with rising temperature. This phenomenon can be elucidated by considering the impact of doping on low‐frequency phonons, leading to a reduction in their lifetimes. Despite the temperature‐dependent variations observed in certain thermal conductivities, the overall thermal transport mechanisms in PEDOT‐based materials underscore the intricate interplay between molecular interactions and doping effects, influencing their thermal properties across a range of temperatures.

**Figure 2:**
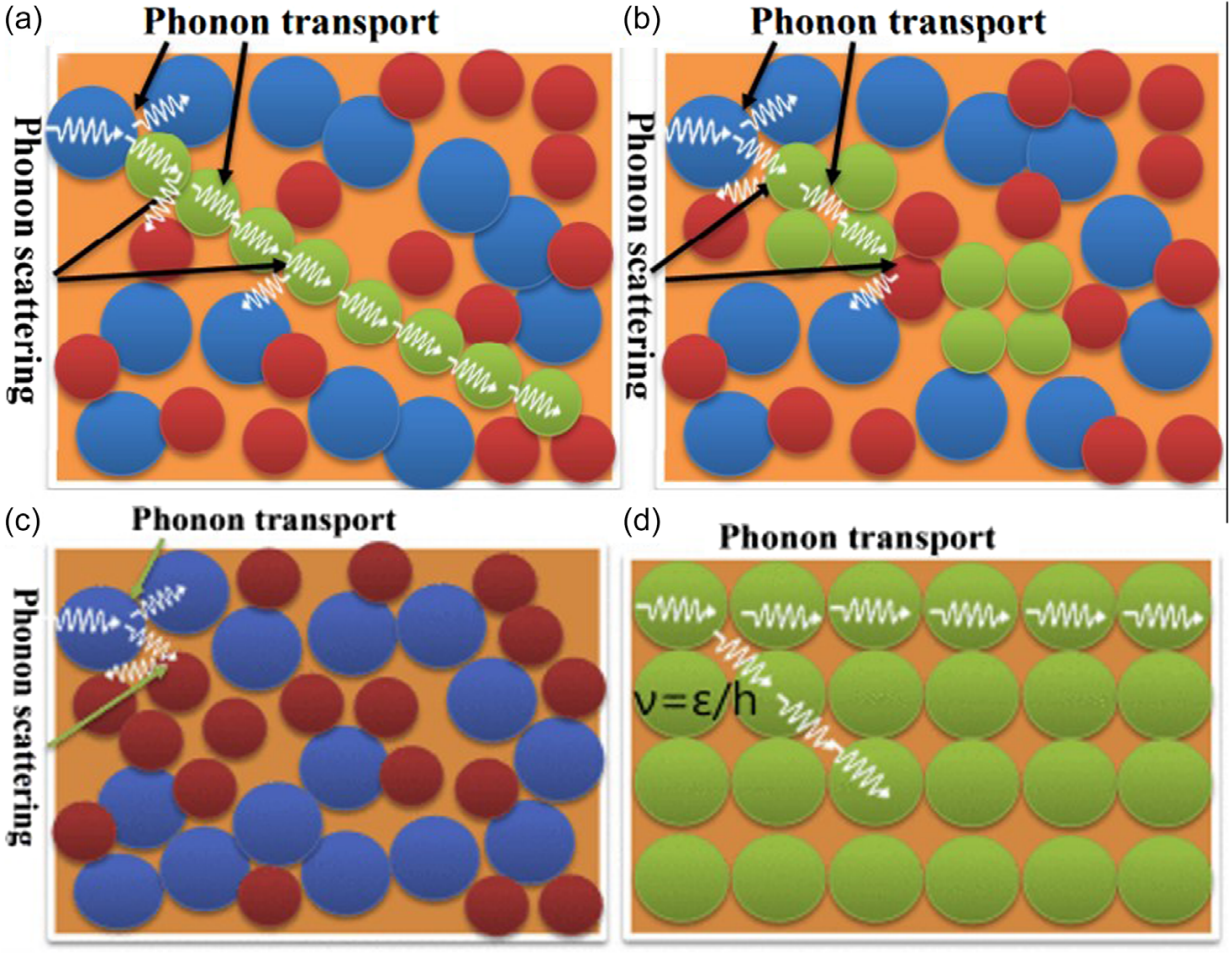
a) Thermal conduction mechanism in crystalline filler/polymer composites with a continuous filler network. b) Thermal conduction mechanism in crystalline filler/polymer composites with a discontinuous filler network. The green balls represent crystalline filler; the others represent polymer atoms. c) Thermal conduction mechanism in amorphous polymers. Different colors and sizes represent different atoms in polymer molecules. d) Thermal conduction mechanism in ideal crystalline structures. The green balls represent crystalline balls. No or less phonon scattering exists. a–d) Reproduced with permission.^[^ ^79^ ^]^ Copyright 2019, Elsevier.

**Figure** **2** discusses the heat conduction mechanism of different conditions, including the heat conduction mechanism of the crystal filling material/polymer composite material in the continuous filling network and discontinuous filling network, the heat conduction mechanism of amorphous polymer, and the heat conduction mechanism in the perfect crystal structure. Doping reduces the thermal conductivity of PEDOT in all three directions at room temperature. The along‐chain thermal conductivity decreases with increasing temperature, while the cross‐plane thermal conductivity shows the opposite trend.^[^ ^22^ ^]^

Moreover, the morphology of PEDOT‐based composites plays a pivotal role in dictating their thermal characteristics. The arrangement and distribution of components within the composite structure significantly influence phonon scattering processes, ultimately impacting the material's overall thermal transport properties. Reducing the dimensionality of PEDOT nanostructures can significantly impact charge transport properties. For instance, in nanosheet samples, the primary source of magnetoresistance is due to surface states with linear energy dispersion. This phenomenon, observed as giant linear magnetoresistance, highlights the potential for PEDOT nanostructures to exhibit unique electrical characteristics.^[^ ^23^ ^]^ The presence of nanoscale interfaces and grain boundaries within the composite structure introduces additional scattering sites for phonons, thereby reducing their mean free paths and impeding thermal transport. Additionally, the orientation and alignment of PEDOT chains within the composite can influence phonon transport pathways, further modulating the material's thermal conductivity. Therefore, a comprehensive understanding of the interplay between dopants, morphology, and phonon transport mechanisms is essential for elucidating the thermal behavior of PEDOT‐based materials and optimizing their thermoelectric performance.^[^ ^24^ ^]^

### 2.3 Sample Preperation

Poly(3,4‐ethylenedioxythiophene) (PEDOT) is synthesized through the oxidative polymerization of the monomer 3,4‐ethylenedioxythiophene (EDOT) in the presence of an oxidizing agent like iron(III) chloride (FeCl3).^[^ ^25^ ^]^ EDOT is polymerized in the presence of polystyrene sulfonate (PSS) to enhance the stability, dispersibility, and conductivity of PEDOT. This results in an aqueous dispersion of PEDOT, which can be easily processed into thin films.^[^ ^26^ ^]^ To enhance thermoelectric performance, PEDOT is often combined with carbon nanotubes for better conductivity, inorganic nanoparticles like bismuth telluride to reduce thermal conductivity, and metal nanowires to improve both conductivity and flexibility.^[^ ^27^ ^]^

Its composites can also be processed into thin films using various techniques. Spin coating involves dispensing a solution of the material onto a spinning substrate to form a uniform thin film, while drop casting entails dropping the material solution onto the substrate and allowing it to spread and dry naturally.^[^ ^28^, ^29^ ^]^ To enhance thermoelectric performance, secondary doping and post‐treatment processes are employed. Secondary doping introduces additional dopants into the PEDOT solution to alter its morphology and enhance charge transport.

For practical thermoelectric applications, the synthetic approaches of thermoelectric nanomaterials should be scalable, high‐quality, and low‐cost with tunable thermoelectric properties, able to form dense compacts for machining and device integration, demonstrate enhanced ZT over bulk materials, and exhibit high thermal stability for extended periods.^[^ ^9^ ^]^ Moreover, mechanical stress from the contact with the heat source and thermal stress from temperature gradients reduce thermoelectric module reliability, so it is essential that thermoelectric materials not only have high ZT values but also possess good mechanical properties.^[^ ^30^, ^31^ ^]^

### 2.4 Doping

Doping adds charge carriers, either positive or negative, into the polymers, enhancing their conductivity.^[^ ^32^ ^]^ P‐type doping involves oxidation and creates positively charged polymers, while n‐type doping involves reduction and creates negatively charged polymers.^[^ ^33^ ^]^ These charge carriers are scattered throughout the polymer chains, which enhances electrical conductivity.^[^ ^34^ ^]^ A fundamental explanation of the doping effect involves the removal of electrons from the highest occupied molecular orbital (HOMO) in the valence band (oxidation) or the addition of electrons to the lowest unoccupied molecular orbital (LUMO) in the conduction band (reduction). This process of oxidation and reduction generates charge carriers in the form of polarons (radical ions), bipolarons (dianions or dications), or solitons within the polymer structure.^[^ ^35^ ^]^ Conductive polymers are classified into degenerate and non‐degenerate types based on their ground‐state bond structures, with degenerate polymers having similar ground‐state structures and non‐degenerate polymers having distinct structures with different energies, such as benzenoid (lower energy) and quinoid (higher energy).^[^ ^34^ ^]^ PEDOT:PSS films typically have low thermoelectric performance and generally require secondary doping and post‐treatment to improve their conductivity. Secondary doping involves adding a dopant to the PEDOT:PSS aqueous solution to alter the morphology of PEDOT:PSS, thereby enhancing charge transfer. PEDOT exhibits excellent Seebeck effect and conductivity, while PPy demonstrates excellent photothermal conversion performance in the infrared spectrum. Therefore, combining PEDOT with PPy can develop materials with higher photothermal‐electric conversion capabilities.

The incorporation of dopants induces notable alterations in the material's volume and promotes phonon scattering, consequently resulting in diminished thermal conductivity along the *y*‐direction. Moreover, the thermal behavior of PEDOT‐based materials is intricately influenced by various factors, including the type and concentration of dopants, as well as the structural morphology of the composite. Dopants introduce supplementary scattering mechanisms for phonons, thereby impeding their thermal transport, particularly at lower frequencies where dopants disrupt the propagation of long‐wavelength phonons, thereby leading to an overall decrease in thermal conductivity.

Additionally, the interaction between PEDOT and dopants modifies the material's phonon dispersion relations, thereby affecting the group velocity and mean free path of phonons, which further contributes to the observed decline in thermal conductivity. The interplay between dopants, composite morphology, and phonon dynamics highlights the complexity of thermal behavior in PEDOT‐based materials, underscoring the importance of comprehensive understanding and tailored design approaches for optimizing their thermal performance.^[^ ^24^ ^]^

### 2.5 Pre‐ and Post‐Treatment

The pretreatment of PEDOT:PSS solution and substrates involves first pipetting 10 mL of PEDOT:PSS solution into a small beaker using a micropipette. Subsequently, the PEDOT:PSS solution is filtered using a vacuum‐assisted filter to remove large particles from the aqueous PEDOT:PSS solution. After filtration, the filtrate is placed in a vacuum chamber for 30 min to remove bubbles. The substrates commonly used in the experiment are silicon dioxide (SiO_2_) substrates and polyimide (PI) substrates. To avoid affecting the performance of the thermoelectric thin film, the silicon dioxide substrates and PI substrates need to be precleaned before use. They are sequentially ultrasonically cleaned multiple times for 20 min each using a series of solutions including a 5 wt% detergent, ethanol solution, deionized water, acetone solution, and isopropanol solution to remove surface organic matter and dust. The cleaned silicon dioxide substrates and PI substrates are then stored in a volatile ethanol solution.

Both the PEDOT:PSS solution and the mold require pretreatment. Prior to preparing the PAM/PEDOT:PSS flexible composite film, the PEDOT:PSS needs to undergo pretreatment using vacuum filtration techniques, followed by placing it in a vacuum chamber for 10 min to remove bubbles. As for the mold, a hydrophobic material such as polytetrafluoroethylene (PTFE) is preferred. PTFE possesses excellent heat resistance, capable of operating within the range of 180–260 °C for extended periods, meeting the heat resistance requirements during film drying. In addition, PTFE demonstrates exceptional resistance to acids, alkalis, and a wide range of organic solvents, rendering it highly corrosion‐resistant against all types of organic solvents, including those commonly used in the production of composite film raw materials and cleaning processes. Its remarkable resistance to chemical degradation ensures the integrity of the mold, even when exposed to harsh chemical environments, thereby prolonging its lifespan and maintaining its efficacy in film fabrication processes. Moreover, the unique hydrophobic nature of PTFE enables the effortless release of viscous hydrogels from the mold, streamlining the demolding process and enhancing production efficiency. Given these advantageous properties, it is crucial to conduct thorough precleaning of the mold before initiating the fabrication of PAM/PEDOT:PSS flexible composite films, ensuring optimal adhesion and compatibility between the mold surface and the deposited materials, thereby guaranteeing the quality and integrity of the final film product.

**Figure 3:**
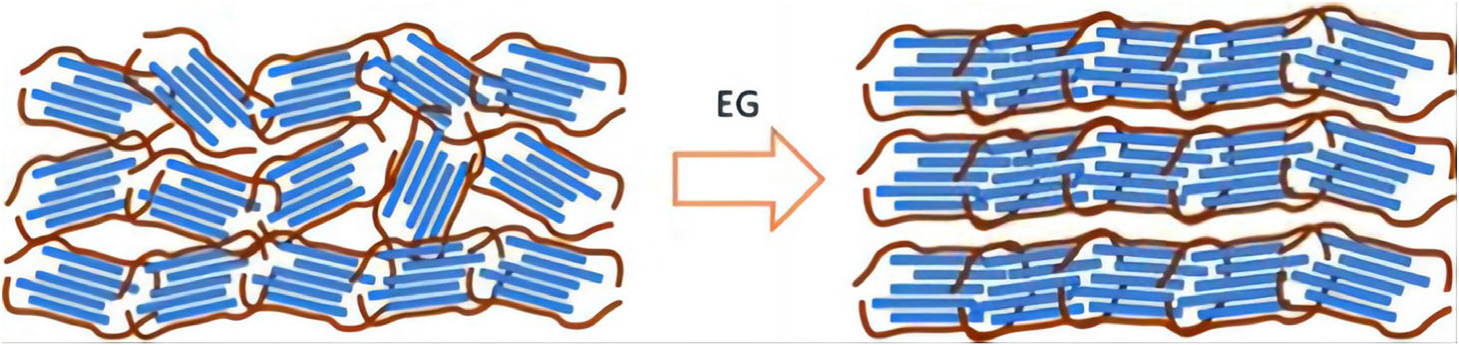
The schematic diagram for the morphological change in the PEDOT: PSS films by secondary doping. Reproduced with permission.^[^ ^80^ ^]^ Copyright 2013, Wiley‐VCH.

**Figure** **3** illustrates the schematic diagram depicting the morphological alteration in the PEDOT:PSS films resulting from secondary doping. Conductive polymers are well‐suited for secondary doping, which allows for the organization of their microstructures in order to enhance the composition and morphology without altering the doping level. Secondary doping can be performed after the initial doping process to simultaneously enhance the Seebeck coefficient (*S*) and electrical conductivity (*σ*). There are many types of pre‐ and post‐treatments to achieve this goal. For example, polar solvent treatment can enhance the order of the polymer microstructure, such as dimethyl sulfoxide (DMSO) which is generally used to process PEDOT:PSS and its *σ* can be improved by a hundred times.^[^ ^36^ ^]^ This is attributed to the high dielectric constant of DMSO and the induced strong screening effects.^[^ ^36^ ^]^ The use of DMSO as a sole solvent treatment has been demonstrated to effectively enhance *σ* of conducting polymers without causing significant changes to the doping level. As a result, the impact on *S* is minimal. This presents an opportunity to improve performance while maintaining the desired *S* value. In addition to treating with polar solvents, post‐treatment methods offer another effective approach for optimizing the microstructure of conducting polymers. Through post‐treatment, the excessive insulating phase can be eliminated through phase segregation, resulting in an increase in *σ*.^[^ ^36^ ^]^ Usually used post‐treatment methods include employing DMSO,^[^ ^37^ ^]^ methanol,^[^ ^38^ ^]^ N, N‐dimethylformamide (DMF) of CuCl_2_,^[^ ^39^ ^]^ formic acid,^[^ ^38^ ^]^ sulfuric acid,^[^ ^40^ ^]^ and then rinse. As for PEDOT:PSS, H_3_SO_4_^+^ and HSO_4_^−^ can interact with positively charged PEDOT^+^ and negatively charged PSS^−^. By inducing phase segregation, part of the PSS is removed, which increases the possibility of PEDOT forming crystals.^[^ ^36^ ^]^ PSS and H_2_SO_4_ are removed by cleaning with excess deionized water. After rational post‐treatment, the PEDOT has been reported with the highest *σ* of up to 4200 S cm^−1^.^[^ ^39^ ^]^ Pre‐treatment is usually combined with post‐treatment, which aims to improve *S* to improve *S* ^*2*^ *σ*, but it may cause potential intermediate damage to the microstructures of conducting polymers.^[^ ^41^ ^]^ In addition to post‐treatment, pre‐treatment methods can also effectively enhance *σ*. For instance, pre‐treatments like nitrogen and argon plasma can be employed to enhance the surface properties of the material, including wettability, hydrophobicity, and dyeability. These pre‐treatment techniques are capable of significantly improving the *σ* of the polymer by modifying its surface characteristics.^[^ ^42^ ^]^ Besides, pre‐treatments also include solvent treatment and oxidant treatment, which impact electrical and mechanical conductive polymers.^[^ ^43^ ^]^ Sequential treatment, involving the combination of secondary doping and doping processes, provides a simultaneous increase in both *S* and *σ*. As previously mentioned, doping enables the adjustment of the oxidation level to enhance the band structure and density of states, thereby improving *S*. On the contrary, secondary doping does not alter the oxidation level of the polymer. By utilizing sequential treatment, it is possible to optimize both *S* and *σ* characteristics effectively.

The PEDOT:PSS underwent a three‐step post‐treatment process involving CH_3_NO, H_2_SO_4_, and NaBH_4_. The experimental procedure was as follows: Initially, the PEDOT:PSS thin film samples were post‐treated with CH_3_NO at room temperature. The dried PEDOT:PSS film samples were immersed in a CH_3_NO solution for 10 min, followed by rinsing the film surface with deionized water to remove excess CH_3_NO. Subsequently, the samples were placed in an 80 °C oven for 10 min to dry, a process referred to as “immersion”. Building upon this, CH_3_NO solution was dripped onto the film samples and heated on a hot plate at 160 °C for 15 min, followed by rinsing the surface with deionized water to remove excess CH_3_NO solution. After washing, the samples were dried in an 80 °C oven for 10 min, termed as “dripping”. The post‐treatment process with CH_3_NO solution proceeded in the sequence of “immersion‐dripping‐immersion‐dripping”. In the second step, the PEDOT:PSS film samples treated with CH_3_NO solution were immersed in an H_2_SO_4_ solution at room temperature for 10 h. Subsequently, the surface was rinsed four times with deionized water to remove excess H_2_SO_4_ solution, and the treated samples were dried in an 80 °C drying oven for 10 min. Before proceeding to the third step, a 200 mL solution of NaBH_4_ with a concentration of 10 mM (i.e., 0.1 mol L^−1^) was prepared. NaBH_4_ powder (0.076 g) was weighed using an electronic balance and mixed with 200 mL of deionized water to prepare the NaBH_4_ solution. The samples treated with CH_3_NO‐H_2_SO_4_ were then immersed in the NaBH_4_ solution at room temperature for 30 min, followed by rinsing the surface four times with deionized water to remove excess NaBH_4_ solution. The treated samples were dried in an 80 °C drying oven for 10 h.

### 2.6 Sample Characterization

Upon the completion of the preparation of PEDOT‐based thermoelectric materials, a comprehensive array of characterization techniques and performance tests are typically employed to meticulously assess their thermoelectric properties. Characterization involves an exhaustive analysis of the material's structure, morphology, and composition, aimed at unraveling its intrinsic characteristics and properties. Concurrently, performance testing entails a qualitative evaluation and empirical testing of the material's functionality and performance under specific conditions, aimed at gauging its usability and applicability. The following are common characterization methods employed: 1) Surface Morphology Analysis: This entails the observation and analysis of the film's surface morphology using advanced imaging techniques such as scanning electron microscopy (SEM) or atomic force microscopy (AFM). These techniques provide valuable insights into surface morphology characteristics, including roughness and texture. 2) Structural Analysis: The film's crystal structure and microstructure are analyzed using techniques such as X‐ray diffraction (XRD) or transmission electron microscopy (TEM). These methods aid in determining the film's crystal phase, crystallographic orientation, and crystallinity. 3) Chemical Composition Analysis: The film's chemical composition is analyzed using spectroscopic techniques such as X‐ray photoelectron spectroscopy (XPS) or Fourier‐transform infrared spectroscopy (FTIR). These methods confirm the presence of specific chemical components and elucidate the bonding configuration within the film. 4) Electrical Property Testing: This involves testing the film's electrical properties, including resistance and conductivity, using specialized equipment such as a four‐point probe resistivity meter or electrical testing system. These measurements provide crucial insights into the material's electrical conductivity and resistive behavior. 5) Thermal Property Testing: The film's thermal properties, including thermal conductivity and Seebeck coefficient, are evaluated using techniques such as thermocouple measurements or thermal flux meters. These tests provide essential information regarding the material's thermoelectric performance and thermal stability.

For example, to characterize PEDOT:PSS film samples, traditional methods such as X‐ray diffractometry (XRD) can be employed, utilizing instruments like the Rigaku SmartLab XRD system. This allows for an in‐depth analysis of the film's composition and phase. Experimental conditions usually entail the use of a copper target, with a tube voltage and current of 40 kV and 15 mA, respectively. The scanning speed is set at 3° min^−1^, covering a range from 2 to 60°. Additionally, microscopic morphology analysis can be carried out utilizing advanced techniques such as AFM and field emission scanning electron microscopy (FESEM), which provide detailed insights into the film's surface structure. To complement these analyses, the thickness of the film samples can be precisely measured using instruments like a profilometer and SEM. These characterization techniques collectively offer comprehensive insights into the structural and morphological attributes of PEDOT:PSS films.

### 2.7 Performance Testing

Following the aforementioned characterization methods, combined with performance testing methods, a comprehensive understanding of the structural characteristics and electrothermal properties of PEDOT‐based film thermoelectric materials can be achieved, providing valuable references and guidance for further optimization and application in practical scenarios. The following are common performance testing methods:

#### 2.7.1 Seebeck Coefficient Testing

The Seebeck coefficient of the film is assessed using a thermocouple or thermoelectric testing system to evaluate its thermoelectric performance. The testing platform for the Seebeck coefficient typically comprises four main components: a humidity control system, a temperature control system, a sample testing platform system, and a data acquisition system. The humidity control system includes a humidifier that regulates steam flow, a humidity detector, and a humidity controller to maintain a constant humidity environment, ensuring the stability of the sample testing environment. The sample testing platform consists of heating and cooling elements, a heat sink, and polyimide. Polyimide, with excellent insulation and thermal stability, is placed below the cooling and heating elements to prevent temperature instability caused by horizontal heat conduction. The temperature control system includes a power supply, heating and cooling elements, a temperature monitoring recorder, a heat sink, and several thermocouples. This system maintains a constant temperature at the hot and cold ends of the testing platform by applying specific power to the heating and cooling elements. Fin‐type heat sinks are attached below the platform to increase the heat dissipation area, achieving rapid equilibrium with the environment. Temperature changes at the hot and cold ends of the testing platform are monitored at distributed temperature measuring points, and the data acquisition system mainly comprises temperature monitoring recorders and digital voltmeters. Based on the temperature difference data obtained from the recorder and the voltage data obtained from the voltmeter, the Seebeck coefficient can be calculated. The formula for calculating the Seebeck coefficient is as follows Equation (4):

(4) $$S=\lim_{\Delta T\to 0}\frac{\Delta V}{\Delta T}$$

In practice, the measurement of the Seebeck coefficient involves determining the potential difference corresponding to a temperature approaching zero. The unit is commonly expressed in mV/K or μV/K. For instance, using a type K thermocouple to indicate temperature, when the temperature difference approaches zero, the V–T curve obtained can be fitted, and the slope of the resulting line represents the Seebeck coefficient of the sample.

#### 2.7.2 Resistance Testing

The film's resistance is measured using a four‐point probe resistivity meter or electrical testing system to evaluate its electrical conductivity and resistive characteristics. For example, conventional four‐probe/square resistance measurements can be performed using an FT‐331 instrument. During the testing process, the system's output current is set to 1 mA, with the uncertainty error being less than or equal to 4%. One set of probes inputs current to the film, while another set of probes detects the potential difference. After passing through the resistivity tester, the resistance R and conductivity *σ* can be calculated using the following Equation (5):

(5) $$\sigma =\frac{ln2}{\pi dR}$$

#### 2.7.3 Power Factor Testing

Power factor testing assesses the film's power factor, which is a crucial parameter for evaluating its overall thermoelectric performance. This testing involves calculating the power factor based on the Seebeck coefficient and conductivity of the film. The power factor is indicative of the material's ability to efficiently convert thermal energy into electrical power, making it a critical metric for thermoelectric device optimization.

#### 2.7.4 Thermal Conductivity Testing

Thermal conductivity testing is essential for evaluating the film's ability to conduct heat efficiently. This test is typically conducted using a thermal flux meter or other specialized thermal testing equipment. By measuring the film's thermal conductivity, researchers can assess its thermal conduction performance, which is vital for understanding heat transfer characteristics within the material. Evaluating thermal conductivity helps in optimizing thermoelectric materials for applications where efficient thermal management is crucial, such as in power generation and waste heat recovery systems.

## 3 Research Progress of PEDOT‐Based Thermoelectric Materials

Compared to other conductive compounds, poly(3,4‐ethylenedioxythiophene) (PEDOT) exhibits a relatively low Seebeck coefficient. However, it compensates for this limitation with its notable strengths in electrical and thermal conductivity, flexibility, and processability. These properties render PEDOT an attractive candidate for various applications, particularly in the development of thermoelectric materials. By leveraging its unique characteristics, PEDOT‐based composites can be tailored to enhance their thermoelectric performance.^[^ ^44^ ^]^

**Figure 4:**
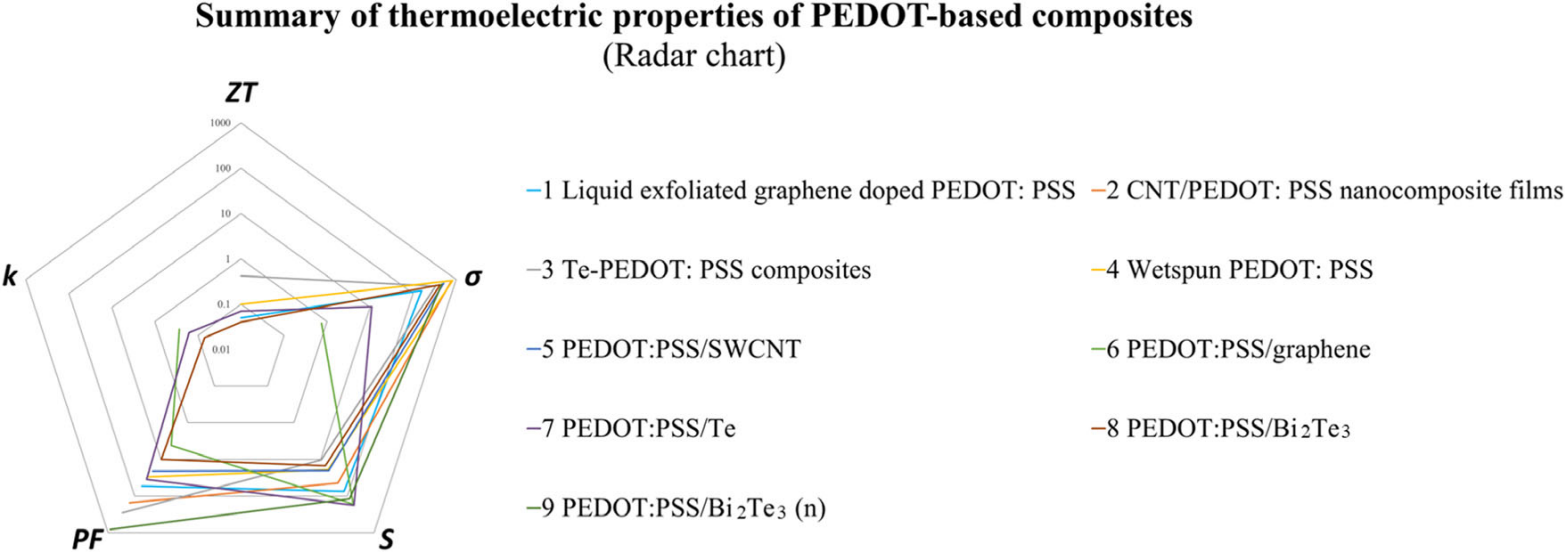
Summary of TE performance of PEDOT‐based composites (Radar chart).

One approach to improve the thermoelectric properties of PEDOT‐based composites is through the design of special morphologies. For instance, incorporating nanostructures into PEDOT matrices can significantly enhance their thermoelectric properties by providing additional pathways for charge carriers and improving electrical conductivity. Furthermore, nanostructuring can effectively reduce thermal conductivity by scattering phonons at the interface, thereby leading to enhanced thermoelectric efficiency. Moreover, exploiting energy filtering effects offers another avenue for enhancing the thermoelectric performance of PEDOT‐based composites. By carefully engineering interfaces within composite materials, it becomes possible to selectively filter out low‐energy charge carriers while allowing high‐energy carriers to pass through unhindered; thus resulting in an enhancement of the Seebeck coefficient. Additionally, harnessing the characteristics of low‐dimensional materials presents yet another opportunity for improving the thermoelectric properties of PEDOT‐based composites. Low‐dimensional materials such as nanowires or nanosheets exhibit unique electronic properties due to quantum confinement effects. Incorporating these materials into PEDOT matrices introduces additional scattering mechanisms for phonons which further reduces thermal conductivity and improves overall thermoelectric performance (**Figure** **4**, **Table** **1**).

**Table 1:** Summary of TE performance of PEDOT‐based composites.

No.	Sample	ZT	*σ* [S cm^−1^]	S [μV K^−1^]	PF [μW m^−1^ K]^−2^]	k [W m^−1^ K^−1^]	References
1	Liquid exfoliated grapheme‐doped PEDOT: PSS	0.05	160	50‐100	53.3	–	[84]
2	CNT/PEDOT: PSS nanocomposite films	–	780 ± 51	43.7 ± 3.3	151 ± 34	–	[85]
3	Te‐PEDOT: PSS composites	0.42	334.68	10.35	284	–	[86]
4	Wetspun PEDOT: PSS	0.1	830	19	30	–	[87]
5	PEDOT: PSS/SWCNT	–	510.6	20.12	20.68	–	[88]
6	PEDOT: PSS/graphene	–	0.74–31.7	165.82–44.75	2.03–6.34	0.24–0.3	[89]
7	PEDOT: PSS/Te	0.07	11	179	35	0.16	[90]
8	PEDOT: PSS/Bi_2_Te_3_	0.04	421	15	9.9	0.07	[91]
9	PEDOT: PSS/Bi_2_Te_3_ (n)	–	470	120	820	–	[92]

In summary, PEDOT‐based composites offer a promising platform for developing high‐performance thermoelectric materials. By optimizing the morphology of these composites and leveraging various mechanisms such as energy filtering effects and interface phonon scattering, significant enhancements in thermoelectric efficiency can be achieved. These advancements hold great potential for applications in energy harvesting, waste heat recovery, and thermal management systems.

### 3.1 Energy Filtering and Quantum Confinement

The energy filtering effect, observed at interfaces between different materials, offers a promising approach to improve *S* without compromising *σ*. By utilizing alternating energy barrier layers, a filter is created that selectively allows high‐energy carriers to pass through, leading to an increased entropy change in carrier transport. Inorganic materials like superlattices and organic–inorganic hybrid systems exhibit significant energy filtering effects, particularly in hybrids with distinct band structures. This effective energy filtering can increase *σ* by 1‐2 orders of magnitude without adversely affecting *S*. However, the underlying principles of energy filtering remain unclear, making it challenging to identify suitable hybrid materials for high thermoelectric performance.^[^ ^36^ ^]^ Furthermore, the attainment of meticulously ordered low‐dimensional structures within organic thermoelectric materials remains a technological challenge, constraining their widespread utilization in various applications. The intricacies involved in precisely controlling the growth of materials at the molecular level underscore the significance of advancing research endeavors in the realm of conducting polymer‐based thermoelectrics. Efforts to engineer ordered low‐dimensional structures entail navigating complex molecular interactions and dynamics, necessitating innovative strategies and precise manipulation techniques. Challenges arise from the inherently disordered nature of organic materials, which often exhibit irregular molecular arrangements and intermolecular interactions. Overcoming these hurdles demands interdisciplinary approaches that integrate principles from materials science, chemistry, and physics to tailor molecular architectures with precision. Emerging methodologies such as molecular self‐assembly, template‐directed synthesis, and bottom‐up fabrication techniques offer promising avenues for orchestrating ordered structures in organic thermoelectric materials. By leveraging these techniques, researchers can exert control over molecular organization and orientation, thereby optimizing charge transport pathways and enhancing thermoelectric performance. Moreover, recent progress in computational modeling and simulation tools has provided invaluable insights into the fundamental principles governing molecular assembly and structure‐property relationships in organic thermoelectrics. By unraveling the underlying mechanisms dictating material behavior, these computational approaches empower researchers to engineer novel molecular architectures with precisely tailored electronic and thermal properties. Consequently, the pursuit of ordered low‐dimensional structures in organic thermoelectric materials emerges as a pivotal frontier in contemporary research endeavors. Through the synergistic integration of interdisciplinary methodologies and the utilization of cutting‐edge technologies, researchers aspire to unlock the full potential of conducting polymer‐based thermoelectrics. This concerted effort aims to transcend existing limitations and catalyze transformative advancements in diverse fields, ranging from energy harvesting and waste heat recovery to novel applications yet to be envisioned. Thus, the interdisciplinary convergence of computational modeling, experimental synthesis, and materials characterization holds promise for driving innovation and realizing the widespread deployment of efficient and sustainable thermoelectric technologies.

### 3.2 PEDOT/ Carbon Nanocomposite Thermoelectric Materials

Carbon nanomaterials, including carbon nanotubes (CNTs) and graphene, exhibit remarkable electrical conductivity and possess unique π–π conjugated structures. These features enable strong π–π interactions with PEDOT, a conductive polymer commonly used in thermoelectric applications. The synergistic interaction between carbon nanomaterials and PEDOT facilitates efficient carrier transport across the material's structure, leading to enhanced electrical conductivity and thermoelectric performance. CNTs, in particular, have garnered significant attention as fillers to improve the thermoelectric properties of PEDOT:PSS composites due to their exceptional electrical conductivity, high aspect ratio, and mechanical robustness. Through precise control of CNT dispersion and loading levels, researchers can tailor the composite's morphology and interface characteristics, optimizing charge transport pathways and minimizing phonon scattering. Additionally, surface functionalization techniques can be employed to further enhance the compatibility and interfacial interactions between CNTs and PEDOT, thereby maximizing the thermoelectric efficiency of the composite material. Overall, the incorporation of carbon nanomaterials offers promising avenues for the development of high‐performance PEDOT‐based thermoelectric materials with tailored electrical and thermal properties.

**Figure 5:**
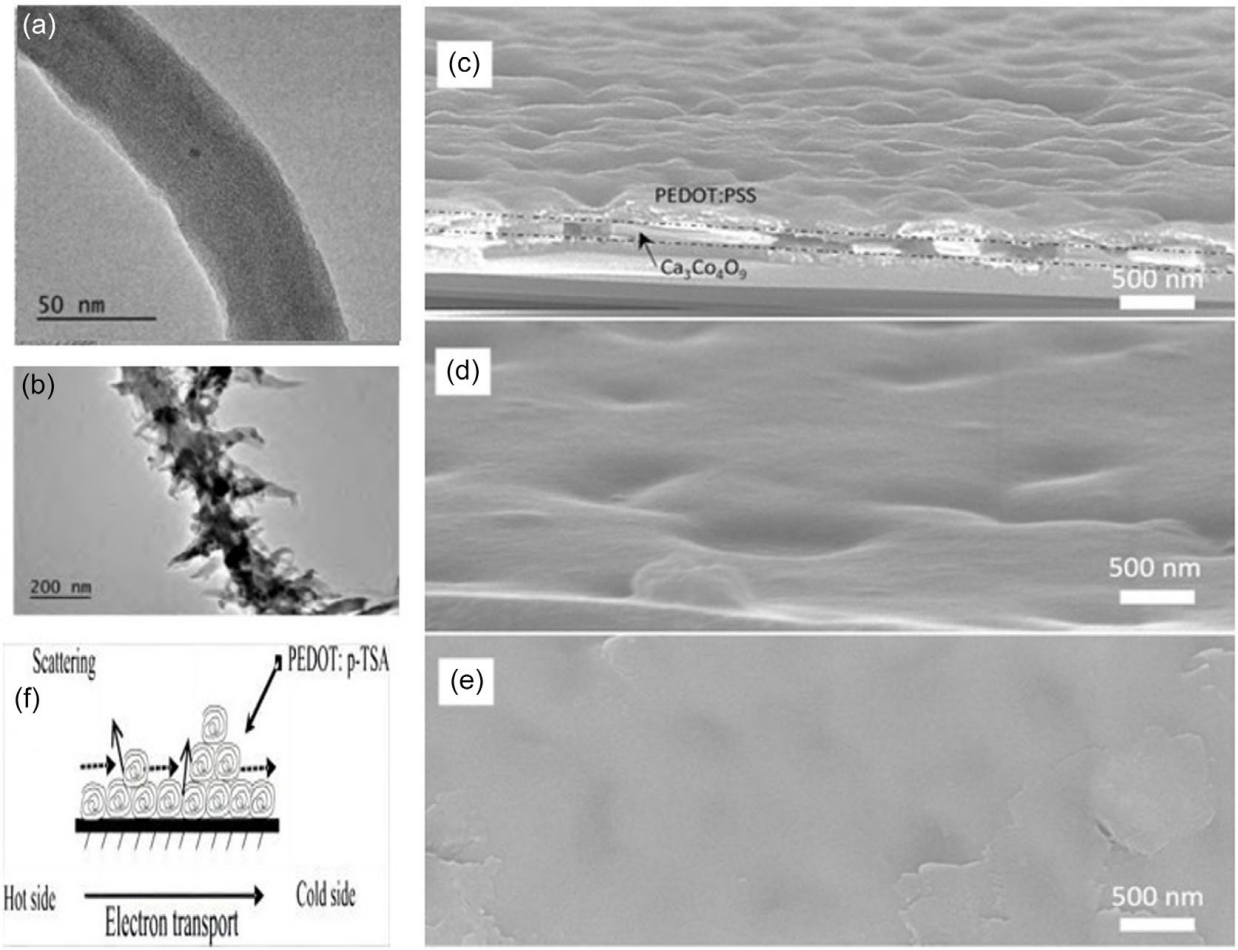
a) PEDOT/SWCNT composite with core–shell structure. Reproduced with permission.^[^ ^44^ ^]^ Copyright 2017, Elsevier. b) Coral‐like morphology of PEDOT/SWCNT composite. Reproduced with permission.^[^ ^44^ ^]^ Copyright 2017, Elsevier. c–e) Cross‐sectional SEM images with tilting 20° of Ca_3_Co_4_O_9_/PEDOT: PSS hybrid films with different solid content 0.25, 0.5, and 1 wt% of PEDOT: PSS dispersions. Reproduced under the terms of the CC‐BY 4.0 license.^[^ ^81^ ^]^ Copyright 2022, The Authors, published by American Chemical Society. f) Schematic diagram of carrier transport of PEDOT: p‐TSA/glass fiber material. Reproduced under the terms of the CC‐BY 4.0 license.^[^ ^82^ ^]^ Copyright 2018, The Authors, published by Scientific Research.

The PEDOT/SWCNT composite with core–shell structure prepared by in‐situ polymerization, due to the 3D network formed by the core–shell structure and the strong π–π interaction, the power factor of the material is significantly improved. When the SWCNT mass fraction reaches 67%, the composite maximum power factor of the material is 157 μW (m K^2^)^−1^.^[^ ^45^ ^]^ In situ chemical oxidative polymerization in inverse microemulsion results in the formation of coral‐like morphology in PEDOT/SWCNT composite thermoelectric materials, which exhibit smaller spacing promoting the hopping transport of local carriers (**Figure** **5**a). Furthermore, the strong interfacial π–π interaction enhances both electrical and thermal conductivity.^[^ ^46^ ^]^

To further improve the dispersion of carbon nanotubes (CNTs) and form a dense network, increase carrier mobility, and enhance the power factor (*PF*) of the composite material, a PEDOT: Tos/sulfonated and acid‐treated SWCNT composite thermoelectric film was prepared through mechanical mixing and its structure is shown in Figure 5b. The acid treatment effectively removed the defect layer on the surface of SWCNTs, and the layered structure and interface interactions of the composite film created additional pathways for carrier transport, leading to a significant increase in conductivity.^[^ ^47^ ^]^ Upon testing, when the mass fraction of acid‐treated SWCNTs reached 35%, the composite film exhibited a maximum *PF* of (119.4 ± 4.3) μW mK^−2^. The thermoelectric properties of PEDOT:PSS/SWCNT and PEDOT:PSS/a‐SWCNT prepared by drop coating method were compared, and the results showed that PEDOT:PSS/a‐SWCNT had higher PF, and the conductivity and The Seebeck coefficient increases with the content of a‐SWCNT.^[^ ^48^ ^]^

Graphene and carbon black serve as alternative additives to enhance the thermoelectric performance of PEDOT, albeit with varying degrees of effectiveness. While single‐walled carbon nanotubes (SWCNTs) have demonstrated notable improvements in thermoelectric conversion efficiency when combined with PEDOT, the addition of graphene and carbon black has yielded less significant enhancements. In particular, graphene, despite its exceptional electrical conductivity and structural properties, has shown limited effectiveness in enhancing the thermoelectric performance of PEDOT when compared to SWCNTs. Studies have revealed that the *PF* of PEDOT:PSS/graphene composites, synthesized via in situ polymerization using chloroform (CHCl_3_) as the solvent and FeCl_3_·6H2O as the oxidant, can reach 45.7 μW mK^−2^, marking a considerable 93% increase over that of pristine PEDOT:PSS.^[^ ^49^ ^]^ In contrast, the incorporation of carbon black into PEDOT matrices has also been explored, albeit yielding mixed results. Composite materials prepared through in‐situ polymerization processes utilizing carbon black as a filler have exhibited varied properties depending on the synthesis conditions. Notably, the resulting PEDOT/carbon black composite materials tend to possess lower electrical conductivity and smaller relative molecular weights compared to pristine PEDOT.^[^ ^50^ ^]^ Despite the limited effectiveness of carbon black in enhancing the thermoelectric performance of PEDOT, efforts to optimize synthesis parameters and material compositions continue. Strategies such as tuning the morphology, size, and distribution of carbon black particles within the PEDOT matrix may hold promise for further improving the thermoelectric properties of the composite material. Additionally, exploring alternative synthesis routes and dopant strategies could provide avenues for enhancing the electrical conductivity and thermoelectric efficiency of PEDOT‐based composites. While graphene and carbon black show potential as additives for enhancing the thermoelectric properties of PEDOT, their effectiveness varies depending on factors such as synthesis method, dopant selection, and material composition. Further research aimed at optimizing synthesis conditions and understanding the underlying mechanisms governing the interaction between PEDOT and these additives is essential for unlocking their full potential in thermoelectric applications.

### 3.3 PEDOT/ Inorganic Semiconductor Composite Thermoelectric Materials

Inorganic semiconductor materials have higher thermoelectric conversion efficiency and power factor, and different preparation methods can significantly improve the thermoelectric performance of PEDOT. At this stage, research on inorganic semiconductor‐modified PEDOT composites mainly focuses on oxide semiconductors, two‐dimensional layered semiconductors, and Te alloys.^[^ ^44^ ^]^

Oxide semiconductors have a high Seebeck coefficient and low thermal conductivity, but due to the low electrical conductivity due to the presence of oxygen in the oxide, the thermoelectric conversion efficiency of their composites is poor.^[^ ^51^ ^]^ The test results of different sizes of ZnO particles combined with PEDOT:PSS show that under the same content conditions, the composite material with large ZnO size has better thermoelectric performance, which is due to the close packing of flakes and the bridging effect of large‐sized ZnO.^[^ ^52^ ^]^ The PEDOT:PSS/ZnO composite film was prepared by spin‐coating multiple layers of 5% DMSO/PEDOT:PSS mixture on the surface of ZnO. It was found that the *PF* of the composite film increased with the increase of the number of PEDOT:PSS spin coatings, which is the multilayer PEDOT:PSS It is more conducive to the formation of a conductive network in the porous structure.^[^ ^53^ ^]^ The changes in morphology and structure of materials under different solid content conditions are shown in Figure 5c–e. The Ca_3_Co_4_O_9_/PEDOT:PSS composite material was prepared by mechanical mixing. The Seebeck coefficient of PEDOT:PSS was limited by Ca_3_Co_4_O_9_. At the same time, due to the poor conductivity of Ca_3_Co_4_O_9_, the transport of carriers between the PEDOT:PSS chains was hindered, resulting in a significant increase in the conductivity of the material.^[^ ^54^ ^]^ Using FeCl_3_ as the oxidant, In_2_O_3_/PEDOT composites with different In_2_O_3_ contents were synthesized by the chemical oxidation method. It was found that when the mass fraction of In_2_O_3_ reached 22%, the maximum *ZT* value of the composite was 0.073 × 10^−4^.^[^ ^55^ ^]^

Two‐dimensional layered materials exhibit high Seebeck coefficients and in‐plane carrier mobility. Among them, M_2_X (M = Cu, Ag; X = S, Se, Te) demonstrates excellent thermoelectric performance at room temperature. Drop‐casting can be employed to fabricate PEDOT:PSS/Ag_2_Se NW composite materials, revealing that the composite material exhibits p‐type semiconductor behaviour when the Ag_2_Se mass fraction is below 50%, and n‐type behaviour when above 50%. At an Ag_2_Se mass fraction of 80%, *S* reaches a maximum value of 51.98 μV K^−1^ and *PF* reaches 183.29 μW mK^−2^. The effects of bending on *S*, *σ*, and *PF* are negligible, signifying good stability and flexibility of the composite film.^[^ ^56^ ^]^ Vacuum filtration allows for the production of Cu_2_S/PEDOT:PSS composite films, resulting in enhanced *PF* of 56.15 μW mK^−2^ at 393 K with a Cu_2_S mass fraction of 10%. The impact of cold pressing on the thermoelectric properties was investigated by measuring the thermoelectric performance of the composite films after being subjected to cold pressing at 1, 2, and 4 MPa. Results indicate that the films exhibit comparable thermoelectric performance after cold pressing at 2 and 4 MPa, attributed to improved film density and conductivity.^[^ ^57^ ^]^ Cu_2_Se/PEDOT:PSS composite films can be prepared on nylon membranes, exhibiting a *PF* of 820 μW mK^−2^ at 400 K. Even after 1000 bending folds, the *PF* remains at 85%, demonstrating excellent flexibility of the film.^[^ ^58^ ^]^

Mxene has the properties of metal and ceramics and has good thermal and electrical conductivity, and the combination of Mxene and PEDOT will generate an internal electric field,^[^ ^59^ ^]^ which can filter low‐energy carriers. PEDOT:PSS/Ti_3_C_2_T_*x*_ composite material can be prepared by drop casting method. After testing, the material has a mass fraction of Ti_3_C_2_T_*x*_ of 16.7%, showing a power factor of 155 μW mK^−2^.

Bi_2_Te_3_ has the best thermoelectric performance at room temperature, and it is also the earliest researched inorganic thermoelectric material. Bi_2_Te_3_ and its alloys have high *S* and *σ*, and PEDOT has low *k*. Combining the advantages of both can effectively improve the thermoelectric properties of composite materials through synergistic effects. Using PS nanospheres as a mask, nano‐Bi_2_Te_3_ is patterned, and different PEDOT/Bi_2_Te_3_ composites are prepared by adjusting the size and spacing of Bi_2_Te_3_ NPs. Since the composites prepared by PS nanospheres with a size of 100 nm have the largest interface The surface area can effectively use the phonon scattering effect and energy filtering effect. When the volume fraction of Bi_2_Te_3_ NP is about 31%, the maximum PF value of about 1350 μW mK^−2^ and the *ZT* value as high as 0.58 is obtained.^[^ ^59^ ^]^ Because the high resistance at the interface between inorganic fillers and PEDOT affects the overall thermoelectric performance, therefore, highly conductive CuTe can be coated on the surface of Bi_0.5_Sb_1.5_Te_3_ to optimize the interface carrier transport of Bi_0.5_Sb_1.5_Te_3_/PEDOT:PSS Performance, the results show that the *σ* of the composite material reaches 2270 S cm^−1^, and the *PF* reaches 312 μW mK^−2^ at room temperature. The thermoelectric device made of this material can generate an output voltage of about 7.7 mV under a temperature difference of about 15 K.^[^ ^60^ ^]^ PEDOT:PSS‐coated Te thermoelectric film prepared by one‐step thermal reduction method, and then prepared PEDOT:PSS/Ag_2_Te composite film by soaking in AgNO_3_ solution. When the concentration of AgNO_3_ solution is 10 mmol, the *S* of the composite film is (55.9 ± 3.3) μV K^−1^.^[^ ^61^ ^]^

### 3.4 PEDOT/ Low Dimensional Metal Composite Thermoelectric Materials

In Figure 5f, a schematic diagram illustrates the carrier transport within the PEDOT: p‐TSA/glass fibre material. This representation provides a visual insight into the underlying mechanisms governing the thermoelectric properties of the PEDOT‐based composite. The unique characteristics of carrier transport in this composite, as depicted in the diagram, play a pivotal role in understanding and enhancing the thermoelectric performance of low‐dimensional metal composite materials. PEDOT can be combined with high‐*σ* metal nanoparticles such as Au, Ag, etc., taking advantage of the inherent high electrical conductivity of the metal nanoparticles and their propensity to form percolation networks within the composite material, thereby enhancing *σ*. Additionally, due to the interface phonon scattering effect between the metal particles and the polymer, *k* is reduced.^[^ ^44^ ^]^

Low‐dimensional metal materials with good dispersion can form more interfaces with PEDOT, and improve the thermoelectric performance of the composite by improving the energy filtering effect and interface phonon scattering effect. AuNPs protected by PEDOT and dodecanethiol (DT) can be prepared into a uniformly dispersed composite film by drop casting. At 50 °C, the mass ratio of PEDOT and Au‐DT NPs is 10^−5^, and the ZT value of the composite film reaches 1.63 × 10^−2^.^[^ ^62^ ^]^

The scale of low‐dimensional metal materials will also affect its distribution. One‐dimensional metal materials have better dispersion than 2D metal materials, so the thermoelectric performance of PEDOT combined with 1D metal materials is better. Thermal effects play a crucial role in the electronic properties of PEDOT/low‐dimensional metal composites. At elevated temperatures, phonon scattering can impact electrons in the bulk bands, causing deviations from linearity and reducing magnetoresistance magnitude. However, surface states, protected by time‐reversal symmetry of the lattice potential, ensure that the thermoelectric performance remains stable even at higher temperatures.^[^ ^23^ ^]^ Using Cu(NO_3_)_2_ as the oxidant, the powder Cu/PEDOT and needle‐like Cu/PEDOT were synthesized by the double in‐situ method. The results show that the needle‐like Cu/PEDOT has better thermoelectric properties compared to Cu nano‐powders when Cu(NO_3_)_2_ is used. When the molar ratio to EDOT is 1, the *PF* of the material is up to 12.47 μW mK^−2^, and the *ZT* value is up to 0.01. This is because the excluded volume of the needle‐like Cu nanoparticles is larger than that of Cu nano‐powders.^[^ ^63^ ^]^

### 3.5 PEDOT/ Multiple Composite Thermoelectric Materials

Combining PEDOT with a variety of nanomaterials can form more interfaces, enhance the interfacial phonon scattering effect of composite materials, form different barrier heights, effectively filter low‐energy carriers, increase *S* and reduce *k*.^[^ ^44^ ^]^

PEDOT‐coated Ag_2_Se/Ag ternary composites can be prepared by in situ polymerization, optimizing the molar ratio of Ag/Se to achieve the maximum power factor.^[^ ^64^ ^]^ Interfacial scattering and energy filtering effects play an important role in the composite, reducing the thermal conductivity and maintaining a proper Seebeck coefficient. Introducing PEDOT:PSS into Ag_2_Se/Cu_*x*_Ag_*y*_Se_*z*_ nanowires can obtain high power factors at different temperatures, and the composite can be fabricated into TE devices with large output voltage.^[^ ^65^ ^]^

Combining PEDOT with inorganic or metal fillers can reduce the flexibility of composites, and adding an appropriate number of polymers at this time helps to obtain thermoelectric composites with ideal mechanical properties. PEDOT:PSS/Ppy/GNS composites can be prepared by in situ polymerization of Ppy/GNS with FeCl_3_·6H_2_O as oxidant and adding it to PEDOT:PSS aqueous solution.^[^ ^66^ ^]^ Due to the hydrogen bond effect and strong π–π conjugation between Ppy and PEDOT, the molecular arrangement is more orderly, which is beneficial to improve the carrier transport and enhance *σ*. When the mass fraction of Ppy/GNS gradually increases, the *σ* of the composite decreases gradually, which is because the transport path of carriers in the composite changes from PEDOT‐Ppy‐GNS to Ppy‐GNS‐Ppy‐PEDOT, and the latter passes through a more interface transmission efficiency is lower. However, with the increase of the number of interfaces, the energy filtering effect and the interface phonon scattering effect are enhanced, making the overall *PF* of the composites tend to rise. When the Ppy/GNS mass fraction reaches 70%, the maximum *PF* is 82.22 μW mK^−2^.

**Figure 6:**
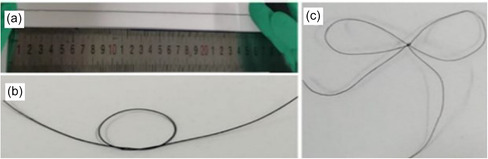
a–c) Digital photos of the PEDOT: PSS/PVA/Te NWs. Reproduced with permission.^[^ ^67^ ^]^ Copyright 2021, Elsevier.

The mechanical properties of PEDOT:PSS/TeNWs were significantly enhanced through a composite approach with PVA using the gelation method. Notably, the PEDOT:PSS/PVA20%/TeNWs35% composite exhibited remarkable improvements, demonstrating a fracture elongation of 12.3% and a power factor (*PF*) of 8.5 μW mK^−2^. When integrated into thermoelectric devices, this composite material yielded an impressive output voltage of 5.03 mV when subjected to a temperature difference of 60 K.^[^ ^67^ ^]^ Furthermore, using electrospinning and in‐situ polymerization, Ag NP/PEDOT:PSS/PVA flexible nanofiber films were produced, demonstrating a maximum *PF* of 1.2 μW mK^−2^. TE devices composed of five of these materials generated an output voltage of 3.43 mV at a temperature difference of 30 K (**Figure** **6**a–c).

## 4 Challenges and Future

### 4.1 Boosting TE Performance

After years of development, significant progress has been made in PEDOT‐based composite thermoelectric materials. However, their large‐scale application remains a challenge. Enhancing thermoelectric performance can be achieved by optimizing four key factors: charge, lattice, spin, and orbital characteristics.^[^ ^28^ ^]^ Hence, enhancing the thermoelectric conversion efficiency of PEDOT‐based composites can be explored from the following perspectives:^[^ ^44^ ^]^ 1) Inorganic thermoelectric materials like Bi_2_Te_3_ and Ag_2_Se are renowned for their exceptional thermoelectric properties. By doping them with additional elements or synthesizing new nanofillers, their performance can be further enhanced. When combined synergistically with PEDOT, these materials contribute to the creation of flexible composite structures boasting elevated thermoelectric conversion efficiencies. The enhancement of electrocatalytic performance by mixing Cu_2–*x*_Se nanorods with carbon materials suggests that similar composite strategies could be employed with PEDOT.^[^ ^68^ ^]^ Incorporating conductive additives could improve electrical conductivity and stability, essential for efficient and durable thermoelectric devices. This strategic integration capitalizes on the unique strengths of both organic and inorganic components, offering promising avenues for advancing flexible thermoelectric devices with enhanced functionality and performance. 2) TE performance can be enhanced by introducing nanostructures and forming new grain boundaries, which induce a potential energy filtering effect leading to enhanced electrical conductivity in the multiscale architecture‐engineered component.^[^ ^69^ ^]^ By compounding PEDOT with various materials or reducing the size of the inorganic component to increase the interface between the two phases, the thermoelectric performance of the composite material can be enhanced through energy filtering and phonon scattering effects at the nanoscale. However, nanowires face practical application challenges as individual wires are only suitable for physical property measurements, while 2D materials suffer from low production yields via PVD or CVD. Nanocrystals, despite their industrial maturity in thermoelectric modules, encounter issues in synthesis, performance enhancement, and stability.^[^ ^70^ ^]^ 3) Current post‐treatment methods for PEDOT‐based composites mainly involve the use of strong polar solvents like DMSO, ethylene glycol, or ionic liquids to remove some PSS from the composite and promote the conformational change of PEDOT from benzene to quinone. Exploring new reagents for post‐treatment offers opportunities to further improve the thermoelectric performance of these composites. 4) The thermoelectric performance of PEDOT is influenced by its oxidation degree and relative molecular weight. Adjusting the preparation process can control the oxidation degree of PEDOT and enhance its thermoelectric performance. Additionally, increasing the relative molecular weight promotes more ordered stacking of PEDOT, leading to a stable macroscopic‐to‐microscopic arrangement that enhances carrier mobility and electrical conductivity without affecting the Seebeck coefficient. This approach can effectively improve the thermoelectric conversion efficiency of the material.

### 4.2 Improving the Design of Conducting Polymers‐Based Devices

**Figure 7:**
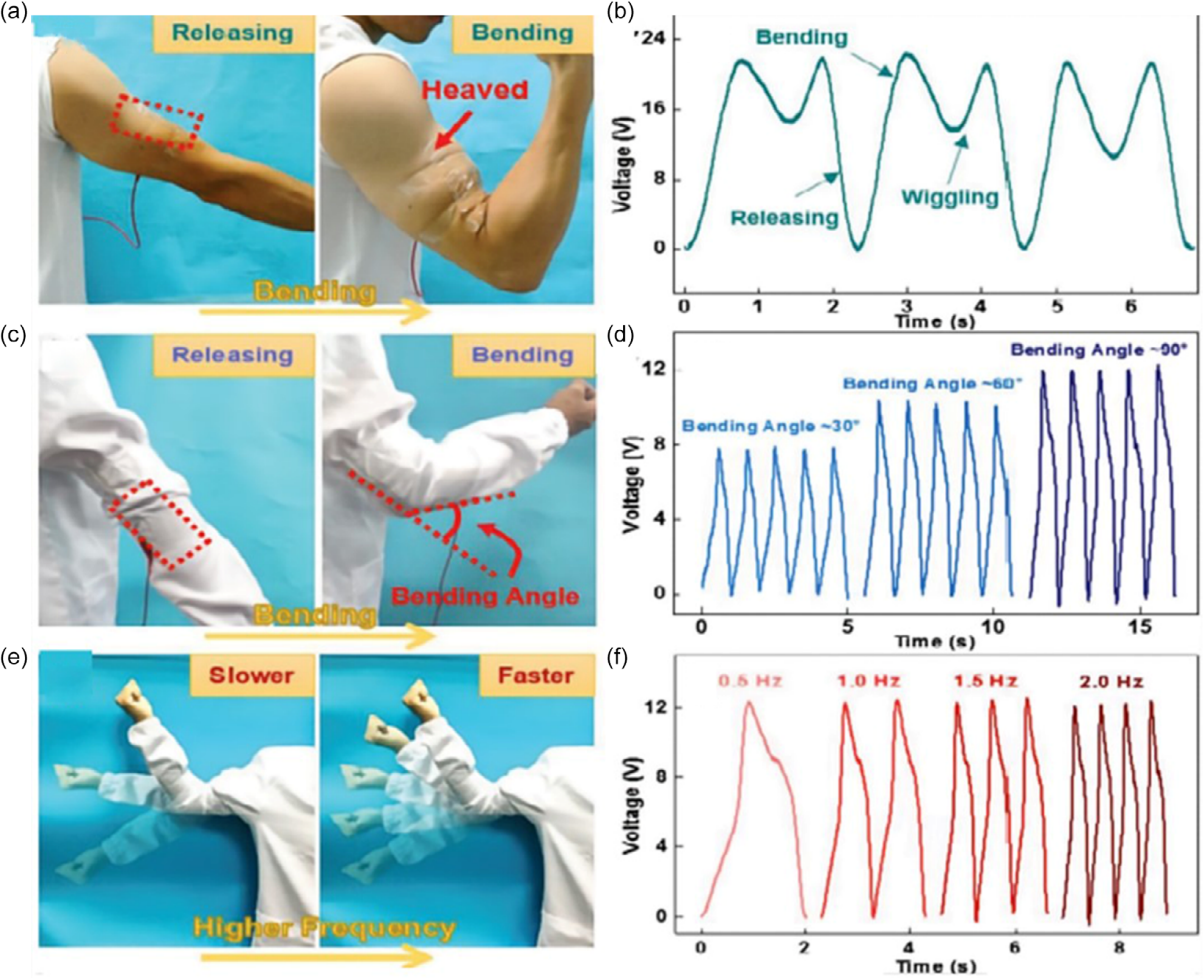
a) Schematic of the active motion sensor mounted on the muscle of the arm for monitoring the motion state. b) Voltage output of the motion sensor during repeated bending and releasing processes. c) Schematic of the active motion sensor fixed at the elbow for monitoring the bending amplitude. d) Voltage output of the motion sensor under different bending angles of the elbow, including 30°, 60°, and 90°, respectively. e) Schematic of the active motion sensor inspecting the bending and releasing process under different frequencies. f) Voltage output of the motion sensor under different bending frequencies, including 0.5, 1.0, 1.5, and 2 Hz, respectively. a–f) Reproduced with permission.^[^ ^83^ ^]^ Copyright 2018, Wiley‐VCH.

The stretchable wearable pulsed triboelectric nanogenerator (WP‐TENG) can be treated as an active sensor for monitoring human movement after being attached to curved human skin, as demonstrated in **Figure** **7**a–f. The figure of merit (*ZT*) of conductive polymers determines the maximum performance of devices, where device structure influences energy conversion efficiency and device volume affects practical application. Therefore, in order to enhance the design of conductive polymer devices, research can be focused on assembly methods such as serial, folding, and stacking configurations, with serial being the most effective.^[^ ^71^ ^]^ To maximize device performance, a serial configuration should be chosen along with increased p–n coupling. Additionally, enhancing electrical conductivity through increased carrier concentration, doping strategies, and composite formation with materials that boost carrier concentration in PEDOT should be explored.^[^ ^72^ ^]^ Construction methods like Y‐shaped or π‐shaped configurations can also be employed to optimize high energy conversion efficiency.

### 4.3 Commercialization

**Figure 8:**
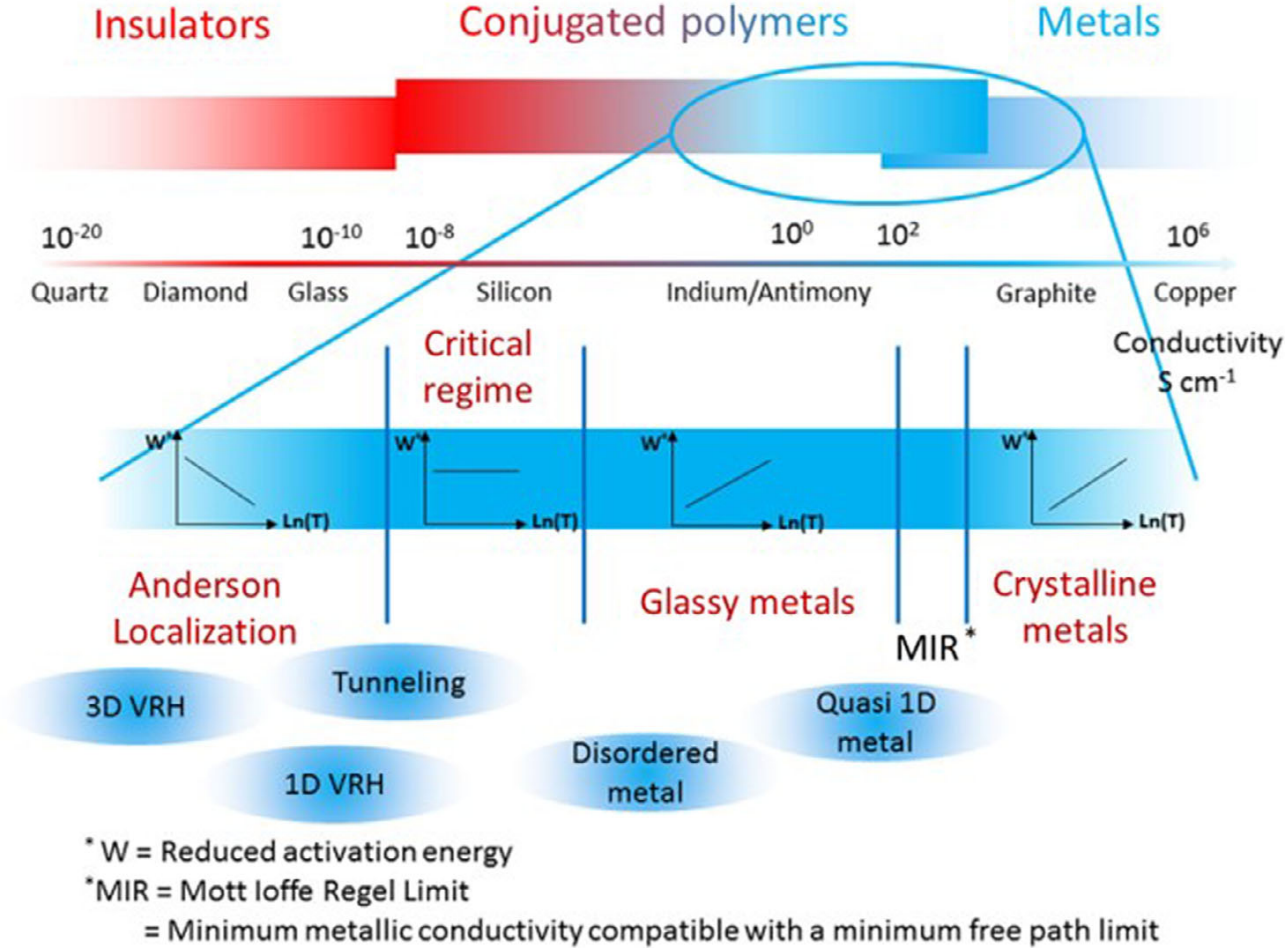
Scheme representing the place of conjugated polymers between metals, semiconductors and insulators. Examples of materials are given as a function of their electrical conductivity. The inset corresponds to conducting polymers and schematizes the different transport regimes that can be found around the critical regime of the insulator to metal transition. Reproduced under the terms of the CC‐BY 4.0 license.^[^ ^10^ ^]^ Copyright 2019, The Authors, published by Elsevier.

**Figure** **8** strategically illustrates the positioning of conjugated polymers amidst metals, semiconductors, and insulators. This scheme, highlighting materials based on electrical conductivity, serves as a visual guide for understanding the versatile applications of conjugated polymers. The inset specifically focuses on conducting polymers, outlining diverse transport regimes around the critical insulator‐to‐metal transition. This depiction provides valuable insights into the potential commercialization avenues and applications of conjugated polymers in electronic devices. Due to the poor conductivity of manganese dioxide and its strong redox coupling with metal electrodes, which compromises safety, conductive polymers were initially adopted as negative electrodes for capacitors to address these two issues.^[^ ^25^ ^]^ PEDOT:PSS was first used as an antistatic layer in photographic films and later as an antistatic coating for cathode ray tubes and various other antistatic applications.^[^ ^73^ ^]^ The high conductivity, good optical transparency in the visible range, compatibility with various deposition techniques, excellent film‐forming ability, and stability of PEDOT‐based materials have led to their commercial application as low‐cost, high‐performance flexible materials. Over the past two decades, the development of various grades of PEDOT:PSS and formulations based on PEDOT has provided possibilities for a wide range of applications, including antistatic coatings and energy conversion and storage devices. PEDOT can be utilized to manufacture transparent conductive electrodes for organic solar cells or light‐emitting diodes.^[^ ^73^ ^]^ The role of PEDOT in the development of electrochemical energy storage devices is also noteworthy due to its low oxidation potential and high charge transfer mobility, although it has the drawback of low mechanical stability during cycling.

Recently, organic materials have emerged in the field of thermoelectrics, and undoubtedly, materials based on PEDOT will play a major role in the development of thermoelectric generators for low‐temperature energy conversion. Organic thermoelectric generators (OTEG) utilize the Seebeck effect for thermal energy conversion. When a thermoelectric material is placed between a heat source and a cold source, thermal energy can be converted into electrical energy. When a temperature gradient is applied across these materials, more electrons flow from the hot side to the cold side. Under steady‐state conditions, the electron concentration gradient is balanced by the internally generated electric field. Nowadays, organic materials have attracted significant interest for such applications, not only because of their low thermal conductivity but also due to their inherent advantages such as low toxicity, lightweight, and good mechanical properties. Moreover, they can be processed from solutions, offering a significant advantage over expensive and often toxic inorganic alloy counterparts.

PEDOT‐based materials also possess characteristics such as electroactivity, electrochromism, thermoelectric performance, and thermal stability. As a result, PEDOT‐based materials have been demonstrated as an actuator material that contracts or expands when subjected to bias and relative humidity conditions or as an materials for thermochromic and electrochromic devices.^[^ ^74^ ^]^ Recently, it have also been applied in medical research, demonstrating potential in cardiac treatment by forming conductive hydrogels that improve tissue conductivity and support heart cell function, offering a versatile platform for treating cardiac injuries.^[^ ^75^ ^]^

In addition to its electronic properties, PEDOT exhibits remarkable compatibility with biological systems and flexible devices, making it highly versatile for various applications. One notable aspect is its ability to undergo changes in doping state through biochemical reactions or electronic signals from devices, allowing for dynamic control of its conductivity. This feature opens up opportunities for tailored interactions with biological environments and adaptive responses to external stimuli. Specifically, PEDOT:PSS, known for its stretchable and conductive nature, has found extensive use in the development of wearable and implantable electronic products. Its flexibility and biocompatibility make it well‐suited for integration into biomedical devices, such as biosensors, neural interfaces, and smart textiles, where conformal contact with biological tissues or curvilinear surfaces is required. Moreover, the tunable doping state of PEDOT:PSS enables precise modulation of its electrical properties, enhancing the performance and functionality of these devices while ensuring compatibility with the physiological environment.^[^ ^76^ ^]^ Commercial applications in soft robotics, human‐machine interfaces, and organic electronic skin are no longer distant prospects.

## 5 Conclusion

In summary, wearable thermoelectric materials based on low‐dimensional PEDOT exhibit significant potential and widespread applications. By producing composites of PEDOT and carbon, inorganic semiconductors, and low‐dimensional metallic materials, we can effectively enhance the thermoelectric properties. In recent years, substantial advancements have been made in the study of PEDOT‐based thermoelectric materials globally, encompassing their fabrication processes, thermoelectric characteristics, and potential applications. Specifically, significant breakthroughs have been achieved in wearable thermoelectric power generators’ creation and commercialization. Going forward, we must enhance the thermoelectric function of PEDOT‐based composites, refine device design, and fortify commercial endeavors. This will facilitate the practical implementation of energy‐efficient, eco‐friendly, wearable smart temperature‐regulating garments and self‐illuminating fabrics. Consistent research and innovation in PEDOT‐based thermoelectric materials are anticipated to make noteworthy contributions within the energy sector and establish a more comfortable and sustainable habitat for humankind.

## Conflict of Interest

The authors declare no conflict of interest.
